# Epidemiology of *Bartonella henselae* infection in pet and stray cats in Croatia with risk factors analysis

**DOI:** 10.1186/s13071-024-06117-8

**Published:** 2024-02-01

**Authors:** Maja Stepanić, Sanja Duvnjak, Irena Reil, Suzana Hađina, Volkhard A. J. Kempf, Silvio Špičić, Željko Mihaljević, Relja Beck

**Affiliations:** 1 Department for Bacteriology and Parasitology, Croatian Veterinary Institute, Savska Cesta 143, 10000 Zagreb, Croatia; 2 Department of Microbiology and Infectious Diseases, with Clinic, Faculty of Veterinary Medicine, University of Zagreb, Heinzelova 55, 10 000 Zagreb, Croatia; 3 Institute for Medical Microbiology and Hospital Hygiene and Consulting Laboratory for Bartonella Infections, University Hospital Frankfurt, Paul-Ehrlich-Straße 40, 60596 Frankfurt, Germany; 4 Department of Pathology, Croatian Veterinary Institute, Savska Cesta 143, 10000 Zagreb, Croatia

**Keywords:** Cat, *Bartonella henselae*, Culture, Multilocus sequence typing, Prevalence, Southeastern Europe

## Abstract

**Background:**

Cats are the primary reservoirs of the bacterium *Bartonella henselae*, the main cause of cat-scratch disease in humans. The main vector of the bacterium is the cat flea, *Ctenocephalides felis*. In southeastern Europe, data are lacking on the prevalence of *B. henselae* infection in cats, the strains of *B. henselae* involved and the risk factors associated with the infection.

**Methods:**

Blood samples collected in ethylenediaminetetraacetic acid-containing tubes from 189 domestic cats (156 pet cats and 33 stray cats) from Zagreb, the capital city of Croatia, and 10 counties throughout Croatia were cultured for *Bartonella* spp. Following culture, bacterial isolates were genotyped at eight loci after using PCR to amplify 16S ribosomal RNA (rRNA) and the internal transcribed spacer region between the 16S and 23S rRNA sequences. Univariate and multivariate logistic regression were used to identify risk factors for *B. henselae* infection in cats.

**Results:**

*Bartonella* spp. was detected in 31 cats (16.4%), and subsequent genotyping at the eight loci revealed *B. henselae* in all cases. Thirty complete multilocus sequence typing profiles were obtained, and the strains were identified as four sequence types that had been previously reported, namely ST5 (56.7%), ST6 (23.3%), ST1 (13.3%) and ST24 (3.3%), as well as a novel sequence type, ST33 (3.3%). The univariate analysis revealed a significantly higher risk of *B. henselae* infection in cats residing in coastal areas of Croatia (odds ratio [OR] 2.592, 95% confidence interval [CI] 1.150–5.838; *P* = 0.0191) and in cats with intestinal parasites (OR 3.207, 95% CI 1.088–9.457; *P* = 0.0279); a significantly lower risk was identified in cats aged > 1 year (OR 0.356, 95% CI 0.161–0.787; *P* = 0.0247) and in cats sampled between April and September (OR 0.325, 95% CI 0.147–0.715; *P* = 0.005). The multivariate analysis that controlled for age showed a positive association with the presence of intestinal parasites (OR 4.241, 95% CI 1.243–14.470; *P* = 0.0119) and coastal residence (OR 2.567, 95% CI 1.114–5.915; *P* = 0.0216) implying increased risk of infection, and a negative association with sampling between April and September (OR 0.379, 95% CI 0.169–0.848; *P* = 0.018) implying a decreased risk of infection. After controlling for the season, an increased risk of infection remained for the coastal region (OR 2.725, 95% CI 1.200–6.186; *P* = 0.012).

**Conclusions:**

*Bartonella henselae* is prevalent throughout Croatia and is a public health threat. Environmental and host factors can significantly affect the risk of infection, and these should be explored in more detail. The presence of intestinal parasites highlights the need to eliminate the flea vector, *Ctenocephalides felis*, as the most effective approach to control infections in cats and humans.

**Graphical Abstract:**

**Figure:**
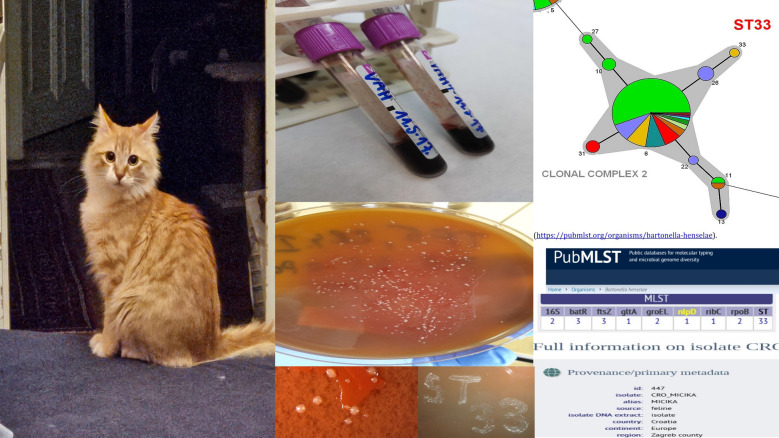

## Background

*Bartonella* spp. are slow-growing alpha-proteobacteria [1], with more than 40 species and 20 *Candidatus* [2–4], of which at least 13 are zoonotic [5, 6]. These bacteria are well adapted to their mammalian animal reservoirs, in which they usually cause long-term intraerythrocytic bacteremia that is mostly asymptomatic [3–5, 7, 8]. Several species of *Bartonella* have been detected in the blood of domestic cats (*Felis catus*), of which *Bartonella henselae* is the dominant infective species. However, cats also serve as natural reservoirs for less common *Bartonella* spp., such as *B. clarridgeiae* and *B. koehlerae*, and as accidental hosts for *B. bovis*, *B. quintana* and *B. vinsonii* subsp *berkhoffii* [3, 5, 9, 10, 15, 61]. *Bartonella* spp. are mainly transmitted through infected blood-sucking arthropod vectors, although other modes of transmission, such as feline blood transfusions, have also been observed [5]. The major competent vector of *B. henselae* is the cat flea, *Ctenocephalides felis* [10–14]. In both cats and humans, infection primarily occurs following a cat scratch, since multiplied bacteria from flea feces contaminate the cat's claws and then enter the injured skin [2, 14, 17]. Humans exposed to infected cats can develop cat-scratch disease [3, 4, 10, 15, 16]. Ticks are also recognized as possible vectors of *Bartonella* spp. [3, 5, 10], but there is currently no consensus on the ability of *Ixodes ricinus* ticks to transmit *B. henselae* to cats [8, 12–15, 70].

The prevalence of *Bartonella* spp. infection in cats appears to vary widely around the world [5, 10, 18], ranging from 1.6% among pet cats in Canada [19] to 62% among stray cats in France [20], as determined by culture, or from 4.7% in stray cats in Greece [64] to 39.9% in sheltered cats in Brazil [60], as determined by PCR. Prevalence therefore depends not only on the diagnostic methods used, which vary in sensitivity [15, 35, 57], but also on the geographic area, characteristics of the cat population and living conditions, all of which appear to strongly influence the risk of feline infection [5, 10, 18]. Frequently published risk factors for *Bartonella* spp. infection in cats include young age and flea infestation [21, 22], living outdoors or as a stray [19–21, 23, 24], warm climate [22] and lack of prophylaxis against ectoparasites [24–26]. In the USA, the risk of cats having bacteria in the bloodstream was 80% lower in cats aged ≥ 13 months than in cats aged up to 6 months (odd ratio [OR] 0.18, 95% confidence interval [CI] 0.05–0.61) [22]; the same lower risk was found in cats that lived strictly indoors compared to outdoor cats (OR 0.18, 95% CI 0.06–0.54) [21]. In Poland, cats from the warmest region of the country were found to have a 2.01-fold higher risk of *Bartonella* spp. infection than cats from cooler regions (OR 2.01, CI 1.16–3.47). Similarly, cats that were not consistently treated against ectoparasites had a 2.02-fold higher risk of bacteremia, with a 95% CI of 1.41–2.92 [26]. The risk for *B. henselae* infection in stray cats in China was reported to be more than double that of client-owned cats (OR 2.283, 95% CI 1.093–4.772) [24]. Additionally, a study in the USA reported that the risk of *Bartonella* spp. infection was threefold higher in cats with fleas than in uninfested cats (OR 2.82, 95% CI 1.1–7.3) [22]. The broad geographic variation in prevalence of *Bartonella* spp. infection in cats and the potentially widespread distribution of risk factors for this infection highlight the need for each country or region to assess these parameters locally in order to guide control and monitoring efforts.

Data are lacking on the prevalence and genetic diversity of *B. henselae* in cats in Croatia and most of the surrounding countries of Central and Southeastern Europe [5, 27]. The bacterium is known to infect cats in Croatia [28–30] and to circulate in humans [31–33]. Therefore, the aim of the present study was to assess the prevalence of *Bartonella* spp. in cats based on multilocus sequence typing (MLST), and to examine the risk factors for such infection.

## Methods

### Sampling of cats

For this study, we analysed blood samples collected in ethylenediaminetetraacetic acid-containing tubes (EDTA-blood samples) between 2014 and 2017 from 189 domestic cats (156 pet cats, 33 stray cats) that had been examined or undergone minor surgical procedures at 20 veterinary clinics at 13 locations around Croatia (nine inland locations, four locations along the coast). The majority of blood samples included in the analysis were collected from apparently healthy cats (*N* = 149), while 40 (21%) cats showed clinical signs.

Veterinarians who agreed to participate in the study were sent a questionnaire that had been specially designed to provide information that could shed light on risk factors for *Bartonella* spp. infections in cats. Cat owners were informed of the purpose of sampling and agreed to provide the requested information on the animals and on the presence/absence of cat-scratch disease in their homes. Age of the cat at the time of blood sampling was recorded by veterinarians based on information provided by the cat owners or based on the veterinarian’s estimate (see Table 2 for data on animal health, treatments, living and environmental conditions, and other data collected by means of the questionnaires and selected for the risk factor analysis).

Venepuncture was performed aseptically, and blood was collected directly into commercial Vacuette tubes (Greiner Bio-One, Kremsmünster, Austria) containing ethylenediaminetetraacetic acid dipotassium salt dihydrate (K2-EDTA) as anticoagulant. Samples were stored frozen until their analysis at the Croatian Veterinary Institute (Zagreb, Croatia) for the present study.

### Culture of *Bartonella* spp. in cat blood

Prior to feline blood inoculation, we conducted initial tests on the culture media using two reference strains of cat-related species (*B. henselae* ATCC 49882 and *B. clarridgeiae* ATCC 700095), purchased from the American Type Culture Collection (ATCC, Manassas, VA, USA). These tests aimed to assess laboratory conditions, growth quality and colony recognition. Since solid media were our first choice, aliquots of each of the 189 samples of thawed and well-mixed cat’s blood were inoculated directly onto the surface of the specific agar medium and the plates rotated to ensure an even distribution. All agar media used included defibrinated sheep or rabbit blood: tryptic soy agar [36], brain–heart agar [37], Columbia agar [20, 38], chocolate agar [22, 39] and previously unexplored esculin-blood agar (No. 2 blood agar base, 5% sheep blood and 0.275 g/l esculin powder, prepared as Koch-sterilized 1% aqueous solution and added to the medium at 27.5 ml per liter). The volume of blood in the sample, which ranged from 0.5 to 3.0 ml, determined how many agar plates per sample could be used; thus, each blood sample was inoculated onto two to five different types of agar media to increase the chances of obtaining isolates.

For a small number of blood samples there was enough blood for additional inoculations, using one or two types of biphasic media consisting of a liquid medium layered on an agar slant in a test tube: tryptic soy agar with tryptic soy broth [1] and brain–heart agar [37] with *Brucella* broth [38]. All media were freshly prepared from dehydrated bases manufactured by Merck (Darmstadt, Germany), with the exception of the *Brucella* broth (BD; Sparks, MD, USA).

Inoculated media were incubated for at least 4–8 weeks at 37 °C in a humid atmosphere containing 8% CO_2_ and checked for bacterial growth twice a week. Agar surfaces were inspected for typical *Bartonella* spp. colonies, which appear as single, hard colonies that are firmly embedded in agar [9, 35]. The isolation rate for each medium was calculated by dividing the number of *B. henselae* isolates obtained on that type of medium with the total number of that medium inoculated with the blood of infected cats.

### Multilocus sequence typing

Primary or subcultured colonies from solid and biphasic media were subjected to multilocus sequencing as follows. Bacterial colonies were mixed with DNA extraction solution containing 180 µl of lysis buffer (“Buffer AL”) and 20 µl of proteinase K from the QIAamp DNA Mini Kit (Qiagen, Hilden, Germany). The mixtures were incubated at 56 °C for 20 min until lysis was complete, then centrifuged at 6000 *g* for 1 min and the supernatant processed using the QIAcube automated DNA extraction system (Qiagen) according to the manufacturer's protocol for blood and body fluids. As an isolation control, 200 μl of phosphate-buffered saline (PBS) was processed in parallel. The isolated DNA from each sample (200 µl) was stored at − 20 °C until PCR testing.

Conventional PCR testing was conducted on aliquots of extracted DNA, including the isolation control sample, using the Pro Flex PCR System (Applied Biosystems, Thermo Fisher Scientific, Waltham, MA, USA) and previously described primers [69] to amplify regions of the 16S ribosomal RNA (rRNA) and the internal transcribed spacer between the 16S and 23S rRNA. Analysis of the 16S rRNA allows differentiation of genotype I (“Houston-1”) and genotype II (“Marseille”) of *B. henselae* [20, 41]. Each 20-µl PCR reaction volume contained 10 µl HotStarTaq Master Mix solution (Qiagen), 7 µl of water free of DNase and RNase (Qiagen), 0.5 µM of each primer (Macrogen, Amsterdam, The Netherlands) and template DNA or PBS (2 µl). During each PCR run, there was both an amplification negative control containing 2 µl DNase/RNase-free sterile water and a positive control containing 2 µl of DNA purified from the reference strain *B. henselae* Huston-1 (ATCC 49882). The thermal cycling conditions were: one cycle at 95 °C for 15 min; followed by 40 cycles of 95 °C for 45 s, 55 °C for 45 s and 72 °C for 45 s; with a final extension at 72 °C for 10 min, followed by a hold at 4 °C. PCR amplicons were visualized on a capillary electrophoresis device (QIAxcel System; Qiagen) and stored at − 20 °C for 1 to 24 months, until sequence typing.

To prevent contamination, we performed the DNA extraction, preparation of PCR reagents, addition of DNA template to PCR reactions, running of the PCR reactions and capillary electrophoresis in separate, dedicated rooms using disposable pipette tips, gloves and aprons.

The DNA amplified from isolates was subjected to sequence typing at eight gene loci, 16S rRNA and genes involved in protein production: *batR* (coding for two-component regulator), *ftsZ* (coding for cell division protein), *gltA* (coding for citrate synthase), *groEL* (coding for heat shock protein 60 chaperon), *nlpD* (coding for cell surface glycoprotein), *ribC* (coding for riboflavin synthase) and *rpoB* (coding for RNA polymerase subunit), as previously described [42, 43]. This widely used typing method has been used to define 37 sequence types of *B. henselae* differing in virulence and zoonotic potential [18, 27, 44, 45]. The DNA was purified using ExoSAP-IT® (USB, Cleveland, OH, USA) according to the manufacturer's instructions, then sequenced by Macrogen. The sequences obtained were used to define alleles and sequence types based on comparisons with the PubMLST database containing all reported *B. henselae* sequence types (https://pubmlst.org/organisms/bartonella-henselae) [27].

### Phylogenetic analysis of sequence types

Phylogenetic analysis was performed by comparing sequences at the eight target genes using BioNumercis 7.6 (Applied Maths, Gent, Belgium) and data in the PubMLST database. The unweighted pair group method with arithmetic mean (UPGMA) was used to cluster the concatenated allelic sequences of all sequence types identified in this study, together with 36 previously reported sequence types, and to construct a “minimum-spanning” phylogenetic tree (MST) showing their relatedness and evolutionary relationships.

### Statistical analysis

Data were processed statistically using STATA 13.1 (StataCorp, College Station, TX, USA). The normality of data distribution was assessed based on plots and the non-parametric Shapiro–Wilk test. Results of statistical tests were considered significant if associated with *P* < 0.05.

Univariate and multivariate logistic regressions were performed to identify risk factors for *B. henselae* infection in cats. Since the dependent variable in our study is binary (*B. henselae*-positive or -negative cat), we used logistic regression also known as binominal logistic regression. Variables that emerged as significant from the univariate analysis were entered step-wise into the multivariate regression. Where appropriate, results were expressed as ORs with 95% CIs.

### Ethics approval

This study was approved by the Committee for Ethics in Veterinary Medicine (640-01/16-17/63, 251-61-01/139-16-2). Cat owners who provided information to assist veterinarians to complete the study questionnaire were informed of the purposes of this research, and they consented for the anonymized information to be analysed and published.

## Results

Blood from 189 cats ranging in age from 5 months to 16 years were sampled at veterinary clinics around Croatia (Table 1). Most of the cats in our sample (*N* = 156, 82.5%) were pets, and the remaining 33 cats were street cats (4.2%) or from shelters (13.2%) (Table 2). *Bartonella* spp. was detected in 31 animals (16.4%, 95% CI 11.1–21.7) from nine of 13 locations, and prevalence at individual locations ranged from 8.8% to 66.7%**.** Infections in animals were not detected at four distant locations, which were spread throughout the country (Table 1).

**Table 1:** Prevalence of *Bartonella henselae* in 189 cats sampled in Croatia by sampling locations and geographical distribution of sequence types

City (sampling location)	Sampled cats (*N*)	Infected cats (*N*)	Cats infected with sequence type (*N*)^a^				
			ST1	ST5	ST6	ST24	ST33
*Inland areas*							
Bjelovar	10	1	0	0	0	1	0
Cestica	6	1	0	1	0	0	0
Jastrebarsko	23	7	1	4	1	0	1
Osijek	13	0	0	0	0	0	0
Velika Gorica	17	0	0	0	0	0	0
Vinkovci	2	1	1	0	0	0	0
Vukovar	8	0	0	0	0	0	0
Zabok	3	2	0	2	0	0	0
Zagreb	57	5	0	4	1	0	0
*Coastal areas*							
Dubrovnik	9	1	Unidentified (see Results)				
Pula	20	8	2	2	4	0	0
Rijeka	10	5	0	4	1	0	0
Split	11	0	0	0	0	0	0
* Total* *N*^b^	189	31	**4**	**17**	**7**	**1**	**1**

^a^The number of each sequence types (STs) among the 30 complete multilocus sequence typing profiles; the total number of each ST is in bold

^b^Prevalence in all cities: 16.4% (95% confidence interval 11.1–21.7%)

**Table 2:** Univariate analysis to identify risk factors for *B. henselae* infection in cats (*N* = 189)

Risk factor	Category	*B. henselae* status,* N* (%)		OR (95% CI)	*P*
		Yes (*N* = 31)	No (*N* = 158)		
Sampling location (city)				1.129 (1.021–1.249)*	0.007*
Age (continuous variable)				0.985 (0.974–0.997)*	0.0047*
Age, months (categorical variable)	0–12	19 (25)	57 (75)	13–36 versus 0-12: 0.517 (0.221–1.208)	0.0247*
	13–36	10 (15)	58 (85)	37–72 versus 0-12: 0.231 (0.050–1.065)	
	37–72	2 (7)	26 (93)	≻ 73: 1	
	> 73	0 (0)	17 (100)	≻12 versus 0-12: 0.356 (0.161–0.787)*	
Breed	Crossbred	30 (16)	155 (84)	1.722 (0.173–17.120)	0.6397
	Purebred	1 (25)	3 (75)		
Sex	Male	17 (22)	60 (78)	0.504 (0.232–1.097)	0.0814
	Female	14 (13)	98 (88)		
Clinical signs	No	28 (19)	121 (81)	0.350 (0.101–1.218)	0.0877
	Yes	3 (7,5)	37 (92,5)		
Antimicrobials	No	30 (18)	136 (82)	0.206 (0.026–1.589)	0.0966
	Yes	1 (4)	22 (96)		
Ectoparasites observation	No	16 (13)	104 (87)	1.806 (0.830–3.928)	0.1340
	Yes	15 (22)	54 (78)		
Fleas	No	17 (13)	109 (87)	1.832 (0.837–4.011)	0.1275
	Yes	14 (22)	49 (78)		
Ticks attachment	No	29 (16)	154 (84)	2.655 (0.465–15.175)	0.2563
	Yes	2 (33)	4 (67)		
Mange mites	No	30 (16)	155 (84)	1.722 (0.173–17.121)	0.6397
	Yes	1 (25)	3 (75)		
Ectoparasite prophylaxis	No	18 (17)	85 (83)	0.841 (0.386–1.833)	0.6635
	Yes	13 (15)	73 (85)		
Feline immunodeficiency virus test	Negative	6 (18)	28 (82)	1.167 (0.110–12.381)	0.8995
	Positive	1 (20)	4 (80)		
Feline leukemia virus test	Negative	7 (21)	27 (79)	1	0.4808
	Positive	0 (0)	2 (100)		
Vaccination against viral disease^a^	No	28 (18)	126 (82)	0.422 (0.121–1.476)	0.1669
	Yes	3 (9)	32 (91)		
Intestinal parasites^b^	No	25 (15)	147 (85)	3.207 (1.088–9.457)*	0.0279*
	Yes	6 (35)	11 (65)		
Dermatophytes^c^	No	31 (17)	151 (83)	1	0.6635
	Yes	0 (0)	7 (100)		
Living conditions	Indoor only	4 (13)	26 (87)	1.330 (0.429–4.121)	0.6216
	Outdoor access	27 (17)	132 (83)		
Residence	Urban	22 (16)	115 (84)	1.094 (0.467–2.562)	0.8364
	Rural	9 (17)	43 (83)		
Ownership	Stray	7 (21)	26 (79)	0.675 (0.264–1.731)	0.4127
	Have owners	24 (15)	132 (85)		
Origin of stray cat	Shelter	2 (25)	6 (75)	0.750 (0.115–4.898)	0.7668
	Street	5 (20)	20 (80)		
Travel beyond residence	No	31 (18)	146 (82)	1	0.1138
	Yes	0 (0)	12 (100)		
Multiple cats in household	No	13 (15)	75 (85)	1.251 (0.574–2.726)	0.5733
	Yes	18 (18)	83 (82)		
Other animals in household	No	30 (17)	143 (83)	0.318 (0.040–2.499)	0.2529
	Yes	1 (6)	15 (94)		
Dogs in household	No	20 (16)	107 (84)	1.154 (0.514–2.588)	0.7289
	Yes	11 (18)	51 (82)		
Contact with wild animals	No	28 (18)	126 (82)	0.422 (0.121–1.476)	0.1669
	Possible	3 (9)	32 (91)		
Cat-scratch disease in household	No	28 (16)	151 (84)	2.311 (0.564–9.479)	0.2340
	Yes	3 (30)	7 (70)		
Sampling season	Oct-Mar	18 (27)	49 (73)	0.325 (0.147–0.715)*	0.005*
	Apr-Sep	13 (11)	109 (89)		
Climatic area	Continent (inland)	17 (12)	122 (88)	2.592 (1.150–5.838)*	0.0191*
	Coast	14 (28)	36 (72)		

*CI* Confidence interval,* OR* odds ratio

*Statistically significant association at *P* < 0.05

^a^Vaccination against feline leukemia virus, feline immunodeficiency virus and rabies

^b^Observation of *Dipylidium caninum* or *Toxocara cati*

^c^Signs of feline dermatophytosis caused by *Microsporum canis*

*Bartonella henselae* isolates grew more frequently on solid culture media (75.5%; 77/102) and less frequently on biphasic culture media (60.0%; 6/10). The best isolation efficiency was achieved on brain–heart agar (87.5%) and Columbia agar (82.4%), followed by esculin-blood agar (80.0%), biphasic tryptic soy agar and broth (80.0%) and chocolate agar (77.3%). The least effective culture media were tryptic soy agar (54.2%) and biphasic brain–heart agar with *Brucella* broth (40.0%). The first colonies appeared in primary isolation after 4 to 56 days of incubation.

All isolates were found to be *B. henselae*, and 30 complete multilocus sequencing profiles were obtained that belonged to five sequence types (STs): ST5 (accounting for 17 of the 30 profiles; 56.7%); ST6 (7 profiles; 23.3%); ST1 (4 profiles; 13.3%); ST24 and ST33 (each 1 profile; 3.3%). It was not possible to identify one isolate (“DUB5”) because we failed to obtain usable sequence data for the *gltA* gene, which meant we could not determine whether it belonged to ST5 or ST24. While four of the sequence types that we detected have previously been reported, ST33 appears to be a new sequence type and was assigned the numerical code 2-3-3-1-2-1-1-2 based on the gene order 16S, *batR*, *ftsZ*, *gltA*, *groEl*, *nlpD*, *ribC* and *rpoB*. This new *B. henselae* isolate was entered in PubMLST under the name CRO_MICIKA and accession ID 447.

The five sequence types were distributed across eight locations in Croatia (Table 1). The three most prevalent sequence types (ST1, ST5 and ST6) were detected in samples collected at both inland and coastal locations. ST5 occurred more often in samples collected at inland locations (64.7%) than in those collected at coastal locations (46.2%). Conversely, ST6 occurred more often in samples collected at coastal locations (38.5% vs 11.8%). Both ST24 and ST33 occurred in samples collected at the inland locations of Bjelovar and Jastrebarsko, respectively. The unidentified isolate DUB5 was detected in a sample collected at a coastal location (Dubrovnik).

**Fig. 1:**
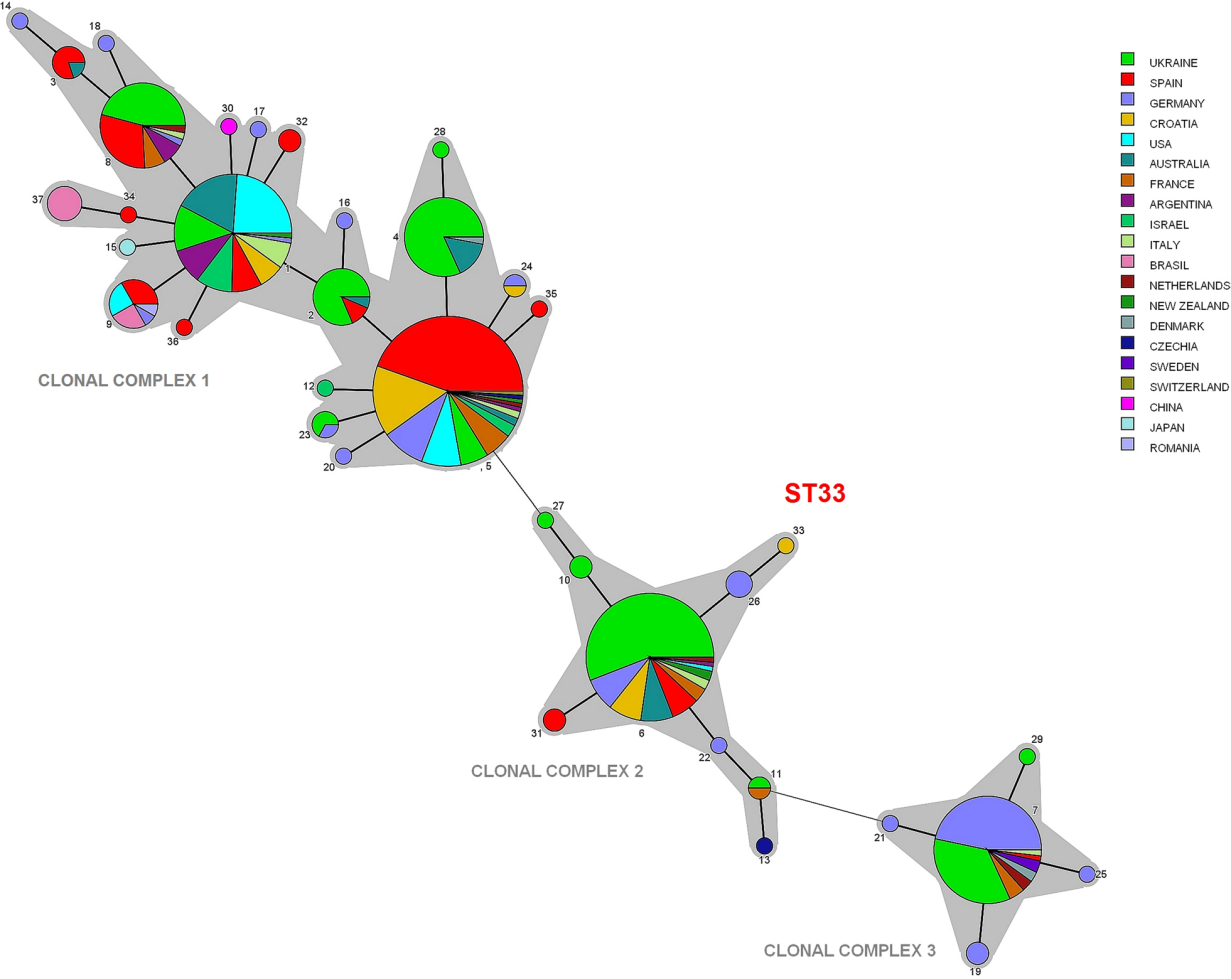
“Minimum-spanning” phylogenetic tree showing putative relationships among *B. henselae* sequence types, based on the unweighted pair group method with arithmetic mean. Isolates are represented as circles, whose size reflects their frequency in the data pooled from the present study and the public database PubMLST, and whose color reflects the country of origin. Numbers refer to sequence types. Thick lines indicate minor allelic differences (in only 1 of 8 genes analysed) and therefore closer relatedness. Thin lines indicate sequence variations in 2 or 3 genes. The sequence type ST33, reported in the present study for the first time, is marked in the middle of the tree

We were able to assign all the sequence types in our study to one of three previously identified clonal complexes of *B. henselae* [43, 44]. The MST (Fig. 1) showed ST33 to be closely related to an isolate of ST26 in Germany [46] and of ST27 in the UK [44]. Adding ST33 to clonal complex 2 increased the cluster from eight to nine members.

In the univariate analysis age > 1 year was associated with significantly lower risk of infection than age ≤ 1 year (OR 0.356, 95% CI 0.161–0.787; *P* = 0.0247), with 19 of 76 cats (25%) in the younger age group showing infection, compared to only 12 of 113 (10.6%) older cats. Risk of infection in coastal regions was 2.6-fold higher than the risk in inland regions (OR 2.592, 95% CI 1.150–5.838; *P* = 0.0191), and risk among cats with intestinal parasites was more than threefold higher than the risk among those without parasites (OR 3.207, 95% CI 1.088–9.457; *P* = 0.0279). The risk of infection was almost 70% lower in cats sampled between April and September (OR 0.325, 95% CI 0.147–0.715; *P* = 0.005) than in cats sampled between October and March. The difference in prevalence between sampling sites (OR 1.129, 95% CI 1.021–1.249; *P* = 0.007), and between cats by age (OR 0.985, 95% CI 0.974–0.997;* P* = 0.0047) was also statistically significant, although weakly associated (Table 2).

We then stepwise included the five factors into the multivariate models and controlled for each factor in turn (Table 3). The association between infection and presence of intestinal parasites remained significant and became even stronger after controlling for age (OR 4.241, 95% CI 1.243–14.470; *P* = 0.0119), but it lost significance after controlling for other factors. The association between infection and coastal residence remained similarly significant after controlling for age (OR 2.567, 95% CI 1.114–5.915; *P* = 0.0216) and season (OR 2.725, 95% CI 1.200–6.186; *P* = 0.012). The association between less frequent infection and sampling between April and September remained significant after controlling for age (OR 0.379, 95% CI 0.169–0.848; *P* = 0.018). The significant association between infection and sampling locations remained weak after controlling for age (OR 1.125, 95% CI 1.014–1.249; *P* = 0.0257).

**Table 3:** Multivariate analysis to identify independent predictors of *B. henselae* infection in cats (*N* = 189)

Models (controlled variables)	OR (95% CI)	*P*
*Model 1 (age as categorical variable)*		
Intestinal parasites	4.241 (1.243–14.47)*	0.0119*
Sampling location	1.125 (1.014–1.249)*	0.0257*
Sampling season Apr-Sep	0.379 (0.169–0.848)*	0.018*
Coastal area	2.567 (1.114–5.915)*	0.0216*
*Model 2 (season)*		
Intestinal parasites	2.765 (0.9–8.381)	0.034*
Sampling location	1.102 (0.984–1.232)	0.194
Coastal area	2.725 (1.20–6.186)*	0.012*
*Model 3 (climatic area)*		
Intestinal parasites	3.029 (0.974–9.422)	0.044*
Sampling location	1.074 (0.925–1.248)	0.345
*Model 4 (sampling location)*		
Intestinal parasites	7.011 (0.863–56.971)	0.0337*

*CI* Confidence interval,* OR* odds ratio

*Significantly associated ORs indicating independent risk factors at *P* < 0.05

## Discussion

We detected a prevalence of *Bartonella* spp. of 16.4% in 189 domestic cats in Croatia using the culture method, similar to the 17% reported for domestic cats in Auckland, New Zealand and to the 16.5% reported for domestic cats in Paris, France [23, 47], but less than the 39.5% reported for pet and stray cats in northern California State, USA [21]. Similar to the studies in New Zealand and the USA, we detected only *B. henselae* in our study animals, while the study in France also detected a small prevalence of *B. clarridgeiae*. Detection of *B. henselae* as the sole species in present study is not surprising since other *Bartonella* spp. associated with cats, such as *B. clarridgeiae* and *B. koehlerae*, are less common and challenging to isolate by culture [3, 5, 9, 10, 20, 22].

The relatively low prevalence of *Bartonella* spp. in our study may reflect, in part, that > 80% of the cats in our sample were pets living with owners and, therefore, more likely to receive better nutrition and flea treatment [24, 61]. In comparison, a study in Spain involving a sample similar to that in our study, with 87% of pet cats living with owners in the former study, reported a low prevalence of *Bartonella* spp. of 7% [38]. Other European studies that sampled cats brought to veterinary clinics reported an even lower prevalence, such as 2.2% in Germany and 5.8% in the UK [44, 46]. In contrast, studies involving higher proportions of stray cats than that in our study reported a higher prevalence, such as 22.6% in Denmark [48] or 28.1% in Turkey [49], while studies of only stray cats in France reported a prevalence exceeding 50% [20, 50].

Although access to the outside world intensifies contacts with other cats and ectoparasites, neither status as a pet versus stray nor as indoor versus outdoor living appeared to significantly affect the risk of infection in our sample. In contrast, studies in several other countries have identified one or both of these as risk factors [18, 21, 25, 26, 53, 62]. We also failed to detect an association between infection and flea infestation [22, 23, 49, 60], or lack of prophylactic flea/tick treatment [24–26, 61], which could also be the result of more appropriate care when cats have owners.

Our analysis among cats in Croatia detected several factors as being associated with higher risk of infection that have also been reported in several other countries. We found a higher infection risk among cats aged up to 1 year, which is consistent with a greater risk among younger animals reported in previous studies [21, 22, 36, 49, 50]. The exact reasons behind the increased susceptibility to infections of kittens and young cats remain unclear. In addition to a relatively weaker immune systems in young animals, there is evidence that fleas have a preference for young cats [71]; in addition, there have been recent suggestions of transmission from mothers to offspring [72]. Furthermore, recent studies have shown that antibodies provide sufficient protection against *Bartonella* spp. by preventing their attachment to red blood cells [73], but also by long-term prevention of reinfection with the same strain [74]. This supports earlier hypotheses that younger animals could lack the protection due to their undeveloped antibodies [15, 21, 50], although more verification of this possibility is required.

Our finding that cats from coastal regions have a greater risk of infection than those from inland areas is consistent with the higher prevalence of *Bartonella* spp. in warmer areas. For example, in the USA, prevalence of *Bartonella* spp. in cats was significantly higher in California and Florida than in Chicago, Washington DC or Michigan [22, 56]. In eastern Poland, prevalence of *Bartonella* spp. in cats was found to be substantially higher in the warmer Subcarpathian region than in cooler areas [26]. These observations may reflect the fact of warmer habitats being more hospitable for the fleas that may carry the bacteria.

We detected a higher risk of *Bartonella* spp. infection in cats between October and March than between April and September, which mostly overlaps with the known seasonality of cat-scratch disease, which in Croatia usually occurs between August and March or in France between September and April [32]. In the present study, for example, we isolated *B. henselae* from three cats in January, March and October, when members of the same households were also diagnosed with cat-scratch disease [28–30], which fully matches aforementioned seasonal pattern. Since cats are more often indoors when the weather turns cold [32], and because they can remain bacteremic long after infection [32], the chances of infection might be increased during the fall and winter.

Interestingly, none of the 53 cats that were sampled in June, July and August during three consecutive years in our study showed *Bartonella* spp. infection, although fleas are generally most abundant during the summer [71]; this result is consistent with the lower prevalence of infection in the summer months seen in a previous study from the USA [57]. Low infection rates in the summer may reflect an increased use of flea protection products, which can prevent *B. henselae* transmission [58, 59], or perhaps low and currently undetectable levels of bacteremia due to the cyclical circulation pattern in cats [4, 5, 29, 57]. In the southern hemisphere, in Brazil, a significantly higher risk for infection was also found in cats in autumn, after the end of the hot summer, possibly reflecting a persistent activity of fleas or a flea treatment has been missed [60]. Therefore, knowledge of the seasonal nature of human and feline infections may be helpful to clinicians when cat-scratch disease is suspected.

In contrast to these risk factors reported above, which are common to Croatia and several other countries, our study detected one risk factor that has yet to be reported: the presence of intestinal parasites. Such an association is new but not surprising because cats with intestinal parasites are more likely to have the cat flea *C. felis* [63], which also serves as an intermediate host for the tapeworm *Dipylidium caninum* [63, 64]. Indeed, four of the six cats with fleas and *B. henselae* infection in our study also had the intestinal parasites *D. caninum* or *Toxocara cati*. Therefore, the practice of simultaneously treating of cats for external and internal parasites is strongly recommended. The association between intestinal parasites and *B. henselae* infection has not yet been discussed, as we are aware of only two studies that have analysed intestinal parasites as potential risk factors [38, 60]. Endoparasites, particularly in young cats, exacerbate an already weaker immune system and increase the likelihood of *Bartonella* spp. infections. As five of these six cats with fleas and *B. henselae* infection in our study were pets, the worrying implications for public health should be addressed; both *T. cati* and *D. caninum* are parasites of zoonotic importance, with a real possibility of transmission to humans [63, 64]. Since intestinal parasites in our study were detected through visual observation of the perianal area or feces, our findings should be verified and extended in studies based on coprology. These considerations highlight the need to include intestinal parasites when analysing risk factors of *Bartonella* spp. infection in cats.

The most reliable method for identifying active *Bartonella* spp. infection is inoculation of cat blood onto solid culture media, followed by molecular typing of isolated bacteria [3, 15, 34, 35]. The use of agar plates for cultivation of *Bartonella* spp. is generally the preferred diagnostic method due to its ability to yield a sufficient quantity and higher quality of DNA for complex genetic investigations [35, 40], such as MLST. We detected five sequence types of *B. henselae* in the Croatian cats included in our study, consistent with the detection of diverse strains of *B. henselae* in cats in several other European countries [18, 44, 46] and elsewhere in the world [42, 43, 51]. The three sequence types that together explained 93% of infections in our sample (ST5, ST6 and ST1) have also been frequently detected in cats from northwestern and Mediterranean Europe [43, 44].

The most frequent sequence types in our study, ST5 (56.7%) and ST6 (23.3%), were also reported to be the most prevalent in Spain (61.5% and 15.4%, respectively), but ST1 was not detected in cats in the Spanish study despite its detection in human isolates [18]. ST5 has also been reported to be quite prevalent in cats from Italy, France, Greece, Germany and the USA [43, 46, 51], and has also been identified in cats in the UK [44], Australia [43] and Algeria (Africa) [37]. In contrast, ST6 was found to be more prevalent (40.0%) in the UK and Australia [42, 44], and also identified in Italy and the USA [43], while it was not detected in Germany [46].

Although ST1 was the third most prevalent sequence type in our study (13.3%), this subtype is actually the most common feline and human sequence type worldwide, having been detected in Europe, Australia, North and South America, Asia and Africa [37, 39, 42–44, 51–54]. ST1 is quite common among cats from the Mediterranean area of Europe (Italy, Greece) [43, 51] and Asia (Israel) [43], but less prevalent in Germany and the UK [43, 44]. Thus, our detection of ST1 and ST5 seems to fit with a potentially widespread presence of these sequence types throughout the Mediterranean region, which remains to be verified in the future.

The ST24 that we detected was previously reported only in Germany [46] and Algeria [37], while ST33 appears to be novel. Since sequence types are thought to develop locally through clonal evolution [46], it is unclear whether ST33 arose in the inland area of Jastrebarsko, where the infected cat lived during sampling, or in the coastal town of Primošten, where it was previously adopted.

The sequence types in our study did not show obvious geographical clustering within Croatia, with the exception that ST6 was observed to occur more frequently at coastal locations (38.5%) than at inland ones (11.8%). Geographically biased distribution was observed in Spain, with ST6 isolates localized to only one of two regions with a similar climate, making it unclear whether such clustering reflects climate or other causes [18]. In contrast, locational clustering of sequence types was not observed in the UK [44].

We also identified diverse sequence types within relatively small areas (approx. 50 km^2^) of cities, such as areas of the cities of Jastrebarsko (ST1, ST5, ST6 and ST33) and Pula (ST1, ST5 and ST6). Diversity was observed even between cats within the same households in the cities of Jastrebarsko and Rijeka (ST5 and ST6), but also within the same shelter in the city of Pula (ST1 and ST6). Such local diversity may reflect the strains carried by fleas. Since cats by nature tend to move only over a limited area, it is assumed that the transmission of *B. henselae* is also quite local [46, 49]. This possibility may also help explain the detection of nine sequence types in Hannover, Germany [46], which appears to be an example of the greatest variability of sequence types in one city. In contrast, infected cats and humans in Asia contain a smaller diversity of strains, dominated by ST1 [39, 52, 53]. Future studies should examine whether these observations reflect differences in local flea populations that presumably infect the cats. However, we are aware of only one study involving fleas conducted in Spain which has demonstrated fleas as carriers of ST5 [18].

 ST5, as the most frequent sequence type in our study (56.7%), is also the one most frequently associated with cat-scratch disease patients, particularly in Europe [18, 43, 44]. A study in Spain, for example, identified this sequence type in > 50% of infected humans, mostly those with the typical form of cat-scratch disease [18]. In the USA, Lindroos et al. [51] detected ST5 in two cats and in samples from their owners with cat-scratch disease, implying transmission from pets to humans. We also confirmed the presence of ST5 in three cats from households with patients diagnosed with cat-scratch disease [28–30]; although the sequences types were not analysed in the affected families, cat-to-human transmission was strongly suspected. All three cat-owner dyads were typical of cat-scratch disease: the cats were young (5 months, 10 months and 2 years), asymptomatic, and had a history of flea infestation. Both cats and humans were diagnosed in the autumn and winter. Two of the three patients were minors (12 and 16 years old), consistent with the greater prevalence of cat-scratch disease among children and young people [10, 16, 31, 32, 55].

In contrast, the presence of ST1 in Spain appeared to be more associated with clinically atypical *B. henselae* infections in humans, such as endocarditis, fever of unknown origin and hepatic peliosis [18]. ST6 has been linked to cat-scratch disease in Australia [42] and France [43]. However, studies in the UK and Spain failed to detect this sequence type in human patients, even though it was detected in cats [18, 44]. Other members of the cluster to which ST6 belongs (clonal complex 2), including the apparently novel ST33, have only been found in cats, but not yet in humans. This coincides with the knowledge obtained from the older division based on 16S rRNA gene analysis, which confirms that all sequence types from our study (ST5, ST6, ST24, ST33) with the exception of ST1 belong to *B. henselae* genotype II, which has been more frequently detected in cats from Europe, USA and Australia than in humans [18, 21–23, 42, 44, 46, 48, 62]. Whether this suggests a lower virulence of the new ST33 for humans remains to be seen in future studies.

Our study describes, apparently for the first time, the culture of *Bartonella* spp. using esculin-blood agar. Widely used to culture mastitis-causing bacteria, such as staphylococci, streptococci and coliforms in diary animals [65, 66], this medium is also used routinely in our laboratories on milk samples, food of animal origin and as a general-purpose bacteriological medium, as well as in scientific studies [67, 68]. It has also proved to be suitable for primary isolation of the fastidious bacterium *B. henselae* through culturing of feline blood samples [30], which represents a novelty.

## Conclusions

We have detected *B. henselae* in 31 cats (24 pet cats and 7 stray cats) from across Croatia, suggesting that the pathogen is widespread throughout the country, posing a public health threat to humans. The situation may be similar in other parts of Southeast Europe, which should be explored in future work. The association between intestinal parasites and *B. henselae* in cats highlights the need to eliminate the flea vector, *C. felis*, as the most effective approach to controlling infections in cats and humans.

## Data Availability

All data generated or analysed during this study are included in this article and are available from the corresponding authors upon the request.

## Abbreviations

16S rRNA: 16S ribosomal RNA
*batR*: Gene coding for the two-component regulator
CI: Confidence interval
*ftsZ*: Gene coding for the cell division protein
*gltA*: Gene coding for the citrate synthase
*groEL*: Gene coding for the heat shock protein 60 (Hsp60) chaperone
K2-EDTA: Ethylenediaminetetraacetic acid dipotassium salt dihydrate
MLST: Multilocus sequence typing
MST: Minimum spanning tree
*nlpD*: Gene coding for the cell surface glycoprotein
OR: Odds ratio
PBS: phosphate buffered saline
*ribC*: Gene coding for the riboflavin synthase
*rpoB*: Gene coding for the RNA polymerase beta subunit
ST: Sequence type
UPGMA: Unweighted pair group method with arithmetic mean
